# The absence of IL17A favours cytotoxic cell function and improves antigen‐specific immunotherapies in pancreatic adenocarcinoma

**DOI:** 10.1002/ctm2.70442

**Published:** 2025-08-19

**Authors:** Giorgia Tiberi, Alessandro Scagliotti, Claudia Curcio, Ermes Candiello, Paula Ariadna Diez Villegas, Silvia Brugiapaglia, Gianluca Mucciolo, Cecilia Roux, Giancarlo Castellano, Roberta Curto, Maddalena Arigoni, Raffaele A. Calogero, Giuseppina Barutello, Niccolò Bolli, Mauro Giulio Papotti, Giulia Adriani, Francesco Novelli, Paola Cappello

**Affiliations:** ^1^ Department of Molecular Biotechnology and Health Sciences University of Torino Torino Italy; ^2^ Hematology Division Foundation IRCCS Ca' Granda Ospedale Maggiore Policlinico Milan Italy; ^3^ Dipartimento di Oncologia ed Emato‐Oncologia University of Milan Milan Italy; ^4^ Pathology Unit, Department of Medical Sciences University of Torino, AOU Città Della Salute E Della Scienza Di Torino Turin Italy; ^5^ Singapore Immunology Network (SIgN), Agency for Science, Technology and Research (A*STAR) 8A Biomedical Grove Immunos Singapore Republic of Singapore; ^6^ Molecular Biotechnology Centre “Guido Tarone,” University of Torino Turin Italy

**Keywords:** cancer vaccine, humoral response, IL17A, pancreatic cancer, tumour immune microenvironment

## Abstract

**Background and Aims:**

The pancreatic tumour microenvironment (TME) is a complex ecosystem where tumour cells, cancer‐associated fibroblasts and immune cells interact, often in ways that contribute to tumour growth. The role of interleukin (IL17)A in pancreatic cancer progression is now more defined, and it is known to sustain a pro‐tumoural microenvironment and inhibit the immune response. Here, we explore the effect of combining IL17A depletion with a cancer vaccine to enhance anti‐tumour immunity.

**Methods:**

We used genetically engineered mice proficient or deficient in IL17A, and orthotopically injected mice with pancreatic tumour cells depleted or not in IL17A, to examine the vaccine effects on tumour growth and immune responses. Both humoral and cellular immune responses were analysed following vaccination in IL17A‐deficient and control mice.

**Results:**

Mice lacking IL17A—either genetically or through pharmacological depletion—exhibited prolonged survival and smaller tumours, compared to vaccinated controls. Vaccination in IL17A‐deficient mice significantly increased the influx of immune cells, including Natural Killer (NK) and effector/memory CD8 T cells, which displayed higher cytotoxic activity. CD8 T‐cell depletion in these models notably reduced vaccine efficacy, underscoring the essential role of these cells. NK cell depletion in untreated models further demonstrated NK cells’ critical function in controlling tumour growth when IL17A was absent. Overall, IL17A depletion enhanced both antigen‐specific humoral and cellular immune responses, indicating a shift towards a more robust and responsive immune environment.

**Conclusions:**

Our findings reveal that the absence of IL17A in the pancreatic TME reprograms it into a more immune‐supportive environment, favouring the recruitment of effector/memory immune cells upon vaccination. This approach paves the way for novel therapeutic combinations in pancreatic cancer, where IL17A depletion may boost both immunotherapy efficacy and anti‐tumour responses.

## Graphical abstract

This study supports the use of anti‐IL17A as a strategy for pancreatic cancer and potentially other cancer. Indeed, in Pancreatic cancer, IL17A blockade enhances the recruitment and activation of CD8 T cells and NK cells, an effect amplified when combined with a cancer vaccine.

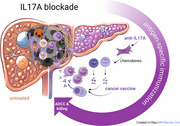

## 1 INTRODUCTION

Despite the encouraging results observed in the fight against cancer, pancreatic ductal adenocarcinoma (PDA) continues to have one of the poorest prognosis, characterised by a 5‐year survival rate lower than 13%.^1^ The high mortality rate is mainly due to the near‐complete absence of symptoms until the advanced stage of disease and its high chemoresistance.^2^, ^3^ In recent years, different immunotherapy approaches have been developed to implement classical anti‐cancer strategies.^4^, ^5^ Unfortunately, the promising results obtained in the treatment of other tumour types are not fully reproducible in PDA.^6^ The main responsible feature of this failure is the strong immune‐suppressive tumour microenvironment (TME) consisting of cancer‐associated fibroblasts (CAFs), T regulatory cells (Tregs), tumour‐associated macrophages, and myeloid‐derived suppressor cells, which together contribute to negatively modulate the action of cytotoxic T lymphocytes in tumour recognition and elimination.^7^, ^8^ Understanding the complexity of this unique TME can enable the design of new different approaches to reshape it and improve the host's anti‐tumour response elicited by both conventional chemo‐ and radiotherapies and more innovative immunotherapy.

Recently, we have demonstrated that interleukin (IL)17A significantly affects CAFs in terms of gene expression and secretome, leading them to sustain the inflammatory response and participate in the recruitment of Tregs, myeloid suppressor cells and granulocytes.^9^ The IL17 family has also been demonstrated to directly affect tumour cells by accelerating neoplastic progression^10^ and invasiveness^11^ or inducing a stem‐like phenotype in tumour cells.^12^ Indeed, genetic depletion of IL17A led to an increased expression of genes related to the T‐cell recruitment and activation by CAFs, together with a general extracellular matrix rearrangement. This was accompanied by an increase in CD8^+^ T cells and M1‐like macrophages in the tumour area and resulted in a longer survival.^9^ This prompted us to assess the therapeutic potential of IL17A depletion in modulating the response to immunotherapeutic approaches and a vaccine strategy. We have developed a DNA‐vaccine targeting alpha‐enolase (ENO1), a moonlight protein with a central role in PDA proliferation and metastasis.^13^ This vaccine significantly prolonged the survival of genetically engineered mice spontaneously developing PDA,^14^ especially when used in combination with chemotherapy,^15^ by inducing a T helper response and the production of antigen‐specific antibodies.

To assess the efficiency of IL17A depletion in enhancing the immune responses elicited by the ENO1‐DNA vaccine, with a particular focus on the cytotoxic response that had previously remained undetected, we employed both autochthonous and transplantable orthotopic mouse models of pancreatic cancer. We also evaluated the antigen‐specific T‐ and B‐cell responses and the general TME composition when IL17A was genetically or pharmacologically depleted. In both models, the stronger T helper (Th)1 response led to an increased cytotoxic ability against tumour cells and higher levels of antigen‐specific antibodies, resulting in longer survival and smaller tumours.

## 2 MATERIALS AND METHODS

### 2.1 Cell lines

The PDA cell line K8484 from Kras^G12D^; Trp53^R172H^; Pdx‐1‐Cre (KPC) mouse was kindly provided by Dr. K. Olive, Columbia University, NY. Pancreatic tumour IL17A^−/−^ cells (KPC/IL17A^−/−^) were isolated from an IL17A^−/−^ KPC tumour mass and purified from stromal cells in our laboratory. Syngeneic murine KPC cell line expressing ovalbumin (KPC‐OVA) and the parental cell line (KPC) were kindly provided by Dr. M. Mazzone (KU Leuven Center for Cancer Biology, Belgium). All cells were cultured in Dulbecco's Modified Eagle Medium (DMEM) supplemented with 20 mM glutamine, 10% fetal bovine serum (FBS) (Gibco, Invitrogen), and gentamicin (40 µg/mL; Fisiopharma).

### 2.2 Mice

The generation of KPC mice has been previously described.^14^ KPC mice (C57BL/6 background) were crossed with IL17A^−/−^ mice (C57BL/6 background, obtained from Dr. Yoichiro Iwakura, University of Tokyo) to generate KPC/IL17A^−/−^. The genotyping of IL17A^−/−^ mice was conducted using the IL17A PCR primers (IL17A universal: 5′‐GTACACCAGCTATCCTCCAGATAG‐3′; IL17A wild‐type: 5′‐AGCACCAGCTGATCAGGACGC‐3′; IL17A mutated: 5′‐GCCATGATATAGACGTGGTGG‐3′). The PCR products were separated on a 1.5% agarose gel. Mice were randomly assigned to control and treatment groups in each experiment with a minimisation strategy based on sex and age, without any exclusion criteria established a priori. Due to the genomic complexity of KPC mice, only one out of eight mice bears both mutated Kras and TP53 and is therefore useful for in vivo experiments. In order to perform a statistical evaluation of the difference between IL17A^−/−^ mice/cells and IL17A^+/+^ mice/cells, (two‐sample *t‐*test or two‐way ANOVA), we calculated a sample size of four, six and nine animals to observe a 40%, 30% and 20% reduction/increase respectively (SD of .2) with a power of 80% and *α* of .05. We also consider increasing the numerosity by one animal/group (total *n* = 6/7/10) to obviate unexpected mortality.

Mice were maintained under specific‐pathogen free (SPF) conditions at the animal facilities of the Molecular Biotechnology Centre ‘Guido Tarone’ and treated conforming to EU, institutional guidelines and Italian Ministry of Health (approval N. 597/2019‐PR and N. 861/2024‐PR). To limit bias in the investigation, only a small number of researchers were responsible for overseeing the animal treatments, and these researchers did not perform the contextual analyses.

### 2.3 In vivo subcutaneous experiments and mouse vaccination

KPC‐OVA cells were treated with mitomycin C (Roche, ref. 10107409001) for 30 min and injected subcutaneously in C57BL/6 IL17A^+/+^ and IL17A^−/−^ mice. Three weeks after this immunisation, both immunised and non‐immunised mice received a subcutaneous injection of 1 × 10^5^ live KPC‐OVA cells in the contralateral flank. Sera were collected prior to the injection of live cells (post‐prime) and at the time of sacrifice (post‐boost). A second cohort of five immunised and non‐immunised mice received 1 × 10^6^ live KPC‐OVA cells and were sacrificed after 1 week for in vitro experiments.

KPC/IL17A^+/+^ and KPC/IL17A^−/−^ mice were vaccinated at 8 weeks of age every 2 weeks for a total of four rounds of vaccination. For vaccination, 50 µg of ENO1‐DNA plasmid was injected into the femoral muscle, previous anaesthesia, followed by 25‐ms transcutaneous low‐voltage electric pulses (amplitude 150 V; interval 300 ms) via a multiple‐needle electrode connected to a Cliniporator (Cliniporator, IGEA). For the orthotopic transplantable model, 1 × 10^5^ syngeneic K8484 tumour cells were injected into the pancreas of C57BL/6 mice (RRID:MGI:7466658). K8484 cells were resuspended in DMEM with growth factor‐reduced Matrigel (BD Bioscience, provided by SIAL) at a 1:4 dilution. Mice were randomly assigned to the different groups: PBS, anti‐IL17A (RRID:AB_10950102, Bioxcell, provided by DBA, Italy), pVax‐ENO1 alone or in combination with the anti‐IL17A.

Neutralising anti‐IL17A antibody was administered twice a week, 10 days after the injection of PDA cells (KPC cells), when the vaccinated groups received the first dose. In this case, mice received a pVax‐ENO1 injection intramuscularly every 10 days for a total of two rounds of vaccination. At 30 days, tumours were weighed, fixed and paraffin‐embedded.

Mice depleted of CD4^+^ T, CD8^+^ T or Natural Killer (NK) cells (RRID:AB_1107636, AB_1107671, and AB_1107737, all from Bioxcell, provided by DBA) received biweekly injections of 200 µg of neutralising antibody until sacrifice, starting 1 day prior to the KPC cell injection for the orthotopic model and at 8 weeks of age for the spontaneous KPC model.

### 2.4 Antigen‐specific Enzyme‐Linked ImmunoSPOT (ELISpot) assay

An ELISPot kit from CTL Europe was used following the manufacturer's instructions. Briefly, 96‐six‐well plates were coated with an anti‐Interferon (IFN‐γ capture antibody overnight at 4°C under aseptic conditions. The following day, 10 µg/mL of recombinant OVA (rOVA) or enolase 1 (rENO1), KPC‐OVA cells or parental KPC cells were employed as stimuli. Phorbol 12‐myristate 13‐acetate (PMA) and Ionomycin (both from Sigma, Italy) were used as positive controls, while splenocytes cultured in only Roswell Park Memorial Institute (RPMI), 10% FBS, and β‐mercaptoethanol (Gibco) represented a negative control. Three hundred thousand splenocytes were seeded at an Effector:Target (E:T) ratio of 20:1. In some cases, ENO1‐specific T cells were expanded in vitro by culturing them in the presence of rENO1 (Sigma) at 10 µg/mL for 1 week. The ELISpot plates were incubated at 37°C in a 5% CO_2_ humidified incubator for 48 h. Subsequently, the plates were processed and analysed with the CTL ImmunoSpot plate reader. The number of antigen‐specific spots was calculated by subtracting the number of spots in the presence of medium only (background) from those evaluated in the presence of stimuli.

### 2.5 Enzyme‐linked immunosorbent assay (ELISA)

A direct ELISA was employed to assess the presence of anti‐ENO1 Immunoglobulins (IgG) or anti‐OVA IgG antibodies in mouse sera collected 2 weeks following the final round of vaccination or at specified time points after immunisation. Half of the plates were coated with 2.5 µg/mL of rENO1 (Sigma) or 3 µg/mL of rOVA (Sigma) diluted in a coating buffer (0.1 M Na_2_CO_3_) and incubated overnight at 4°C. On the following day, plates were washed three times with .05% Tween‐PBS and then incubated at room temperature with 200 µL/well of 4% BSA‐PBS for 1.5 h to block non‐specific binding sites. After two washes, both coated and uncoated wells were incubated with 50 µL/well of sera (1:1000 for ENO1 antibodies and 1:200 for OVA antibodies) diluted in .05% Tween‐1% BSA‐PBS at room temperature for 2 h. After six washes, plates were incubated with 50 µL/well of anti‐mouse Horseradish Peroxidase (HRP)‐conjugated secondary antibodies as follows: anti‐total IgG (RRID:AB_2533947, Invitrogen; 1:8000), anti‐IgG1 (Invitrogen, RRID: AB_10988195; 1:8000), anti‐IgG2b (Invitrogen, RRID: AB_10563452; 1:2000) or anti‐IgG2c (Jackson Immuno Research, provided by Prodotti Gianni; RRID: AB_2338516, 1:2000) for 1 h at room temperature. Then, 50 µL/well of 3,3',5,5' ‐ Tetramethylbenzidine (TMB) solution (Surmodics, provided by Merck) was added for 10–25 min in the dark. The reaction was stopped with 25 µL/well of 2N HCl. The OD values were measured at 450 nm using a VICTOR Nivo Multimode Microplate Reader (Perkin Elmer). The total ENO1 IgG antibody concentrations were calculated by regression analysis using eight 3‐fold serial dilutions of 1 µg/mL 72/1.11 monoclonal antibody (mAb), kindly provided by Dr. Paola Migliorini (University of Pisa, Italy), for a standard curve.

### 2.6 Immunophenotyping

Tumours from KPC/IL17A^+/+^ and KPC/IL17A^−/−^ were dissociated with the Multi Tissue Dissociation Kit II (Miltenyi Biotec) with the OctoMACS tissue dissociator in accordance with the manufacturer's instructions. Monodispersed cells were stimulated with PMA (50 ng/mL), ionomycin (2 µg/mL) and brefeldin A (25 µg/mL, Sigma) for 5 h to analyse the presence of T and myeloid cells. Specifically, cells were washed with .5% BSA‐.01% sodium azide (NaN3)‐PBS and incubated with Fc blocking (Biolegend, provided by Campoverde) for 10 min on ice. The following antibodies and relative rat isotypes were used: anti‐CD4 (RRID: AB_389302), anti‐CD8 (RRID: AB_312750), anti‐CD25 (RRID: AB_893290), anti‐CD11b (RRID: AB_312788), anti‐CD206 (RRID: AB_10896057) and anti‐MHC II (RRID: AB_313322) (all from Biolegend, provided by Campoverde). Following washes, cells were fixed and permeabilised (FoxP3 Fix/Perm Buffer Set, Miltenyi Biotec) and stained for FoxP3 (eBiosciences, RRID: AB_469457), Eomesodermin/Tbr2 (Eomes; Miltenyi Biotec, RRID: AB_2651632), IFN‐γ (Biolegend, RRID: AB_315403), granzyme B (Biolegend, RRID: AB_2687029) and perforin (BD, RRID: AB_2738287). All antibodies were used as suggested by the manufacturers. The cells were then washed and resuspended in PBS. A total of 100 000 events were acquired with an Accuri C6 Flow Cytometer (BD Biosciences) and subsequently analysed using FlowJo X (RRID: SCR_008520, Tree Star from BD Biosciences).

### 2.7 Spatial transcriptomic analysis

A spatial transcriptomics analysis was conducted using the 10X Genomics Visium platform. Pancreata from two vaccinated KPC/IL17A^+/+^ and two KPC/IL17A^−/−^ mice were collected at 16 weeks. Specifically, mice were euthanised per the recommendations provided by 10X Genomics.^16^ Pancreases were rinsed in ice‐cold PBS and embedded in pre‐cooled Optimal Cutting Temperature (OCT) compound, then placed in a bath of cold isopentane in liquid nitrogen until OCT solidification. RNA was extracted from 10‐µm‐thick slides, and only samples with an RNA integrity number greater than 7 were utilised. The Visium slide handling procedure was conducted following the instructions provided by 10X Genomics. Sequencing libraries were run on 2 NextSeq 500 (Illumina) instruments, with pair‐end sequencing (R1: 28 nts, R2: 91 nts), using 400 million cluster cartridges. The deconvolution of cell types and cell type proportions present within each capture location was performed using SPOTlight.^16^ The single cell experiment data from the Tabula Muris Consortium was employed as a reference, comprising >350 000 cells from male and female mice belonging to six age groups, ranging from 1 to 30 months.^17^ The ‘All’ subset of the aforementioned dataset was employed to align it with our Visium slides. The model was trained in accordance with the prescribed workflow.

### 2.8 Histology and immunofluorescence

PDA samples were collected at the indicated time points, formalin‐fixed and paraffin‐embedded. To quantify the tumour burden, 4‐µm‐thick slides were stained with haematoxylin and eosin (H&E). Specifically, the rehydrated slides were immersed in haematoxylin (Agilent Dako) for 20 s, followed by a wash in running tap water. Subsequently, the slides were immersed in Eosin (Agilent Dako) for a further 20 s and washed in distilled, deionised water. The percentage of tumour was quantified using QuPath software (University of Edinburgh) after scanning H&E pancreas sections with the NanoZoomer S60 Digital Slide Scanner (Hamamatsu). For CD4 and CD8 immunofluorescence staining, the rehydrated slides were treated with a 3% hydrogen peroxide aqueous solution for 10 min to inhibit the endogenous peroxidase activity. Antigen retrieval was performed in a microwave oven with an Ethylenediaminetetra‐acetic acid (EDTA) buffer, followed by a 2‐h incubation with primary antibodies: anti‐CD4 (Abcam, RRID:AB_2686917 diluted 1:1000) and anti‐CD8 (eBioscence, RRID:AB_2572861 diluted 1:800) antibodies. Subsequently, slides were washed and incubated with fluorescent‐conjugated secondary antibodies (diluted 1:2000, Alexa Fluor555‐conjugated donkey anti‐rabbit IgG and Alexa Fluor488‐conjugated donkey anti‐rat IgG, RRID:AB_162543 and RRID:AB_2535794, respectively) for 1 h. The nuclei were stained with Hoechst 33342 (1:3000, Invitrogen) for 20 s. Slides were acquired using a TCS SP8 confocal microscope (Leica).

### 2.9 Cytotoxicity assays

Splenocytes from KPC/IL17A^+/+^ or KPC/IL17A^−/−^ vaccinated or non‐vaccinated mice were frozen and stored until usage. PDA spheroids were obtained from K8484 cells, cultured in serum‐free DMEM/F12 medium complemented with 2 mmol/L glutamine (Sigma), penicillin–streptomycin (EuroClone), .4% bovine serum albumin (Sigma), recombinant Epithelial growth Factor (EGF) (20 ng/mL) and beta Fibroblast Growth Factor (bFGF) (10 ng/mL) (both from SIC). After 3 days, the generated spheroids were seeded in a 3D matrix composed of collagen type 1 and Matrigel in a 1:1 ratio as previously described.^18^ The spheroids were then co‐cultured with splenocytes from vaccinated or non‐vaccinated KPC/IL17A^+/+^ or KPC/IL17A^−/−^ mice in a tentative 1:20 E:T ratio. All conditions were repeated with splenocytes isolated from vaccinated or non‐vaccinated and CD8‐depleted mice. The measurement of spheroid total area was conducted using the ImageJ software (RRID:SCR_003070, NIH, Bethesda, MD, USA). The invaded area was normalised to the value observed on day 0 and the resulting fold change was reported in the graph.

In another set of experiments, K8484 cells, were stained with 1 µM of CFSE (Biolegend) diluted in DMEM for 15 min at room temperature in the dark and extensively washed with DMEM 10% FBS before the co‐culture with splenocytes. Two different E:T ratios were assessed (25:1 and 12,5:1). After a 4‐h incubation within a 5% CO_2_ humidified incubator, cells were stained for CD45 (Milteny Biotec), acquired at an Accuri C6 Flow Cytometer and analysed using FlowJo X. The number of CFSE‐positive cells was quantified within the CD45‐negative population.

### 2.10 Degranulation experiment

Splenocytes isolated from OVA‐immunised or non‐immunised mice were cocultured in FACS tubes with filter caps at a density of 2.5 × 10^5^ cells per tube on top of 10^4^ KPC‐OVA or parental KPC cells. After 4‐h incubation within a 5% CO_2_ humidified incubator, cells were stained for CD4, CD8 (both from Biolegend) and CD107 (Miltenyi Biotec). The stained cells were acquired at an Accuri C6 Flow Cytometer and analysed using FlowJo X.

### 2.11 Quantitative real‐time polymerase chain reaction (qRT‐PCR)

RNA was extracted from paraffin‐embedded whole tumours using the PureLink FFPE Total RNA Isolation Kit (Invitrogen), following the manufacturer's instructions. cDNA was generated from 1 µg of RNA using the iScript Reverse Transcription Kit (BioRad). The qRT‐PCR reaction was conducted using PowerUp SYBR Green Master Mix (Applied Biosystems) and specific primer pairs in a CFX Connect RT‐PCR (Biorad). Murine glyceraldehyde‐3‐phosphate dehydrogenase (*Gapdh*) was employed as a housekeeping gene to calculate the relative mRNA expression of the gene transcripts. Primers are listed below:

**Table:** 

Col1a1 forward	5’‐CCCCTGGTCAAGATGGTC‐3’
Col1a1 reverse	5’‐CTCCAGCCTTTCCAGGTTCT‐3’
Acta forward	5’‐GTTCAGTGGTGCCTCTGTCA‐3’
Acta reverse	5’‐ ACTGGGACGACATGGAAAAG‐3’
Postin forward	5’‐AGCAAGCAGGGAAGGAATG‐3’
Postin reverse	5’‐ GAGGCTGAGGAAGATGCTAAAG‐3’
Cxcl10 forward	5’‐ATGACGGGCCAGTGAGAATG‐3’
Cxcl10 reverse	5’‐TCGTGGCAATGATCTCAACAG‐3’
Cxcl11 forward	5’‐CAGCTGCTCAAGGCTTCCTTA‐3’
Cxcl11 reverse	5’‐CTTTGTCGCAGCCGTTACTC‐3’
Ccl5 forward	5’‐TGCTGCTTTGCCTACCTCTC‐3’
Ccl5 reverse	5’‐TCCTTCGAGTGACAAACACGA‐3’
Ccr5 forward	5’‐AAGAGACTCTGGCTCTTGCAG‐3’
Ccr5 reverse	5’‐GAGCTGAGCCGCAATTTGTT‐3’

### 2.12 Statistical analysis

Two‐way ANOVA was used to evaluate the differences in all the experiments when data were normally distributed (checked with Shapiro–Wilk test and QQ plot), except for Kaplan–Meier log‐rank analysis used for the survival of KPC/IL17A proficient and deficient mice, and Student's *t*‐test for ENO1‐specific ELISpot Assay analysis. All tests were performed with GraphPad Prism 10 Software, Inc. (RRID:SCR_002798). Outlier values were calculated by the ROUT method before applying statistical analysis and declared in the figure legends.

## 3 RESULTS

### 3.1 IL17A deficiency enhances antigen‐specific immune responses

**FIGURE 1:**
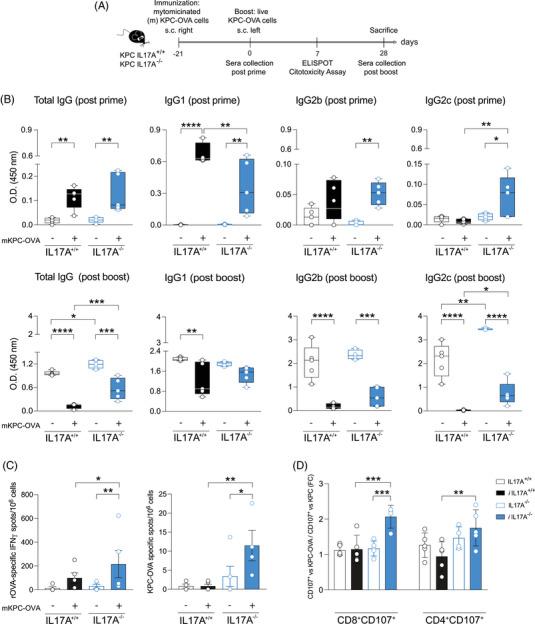
IL17A affects antigen‐specific immune response. (A) Scheme of the experimental model (*n* = 5 mice/group). (B) Evaluation of specific anti‐ovalbumin (OVA) antibodies in the sera of immunised (filled pattern) and not (empty pattern) IL17A^+/+^ (black) and IL17A^−/−^ (blue) mice before the injection of live KPC‐OVA cells (post‐prime, upper row) or at time of sacrifice (post‐boost, lower row). Total IgG, IgG1, IgG2b and IgG2c were measured through a direct enzyme‐linked immunosorbent assay (ELISA). Box and whiskers represent the min to max OD values ± SD; median is indicated; each dot represents a biological replicate. *p ≤ .0332, **p ≤ .0021, ***p ≤ .0002, ****p < .0001 (C) OVA‐specific IFN‐γ‐secreting cells (left graph) were evaluated from splenocytes isolated from immunised (filled pattern) and not (empty pattern) IL17A^+/+^ (black) and IL17A^−/−^ (blue) mice and subtracted of spots obtained in the absence of stimuli. The number of IFN‐γ‐secreting cells was also measured after stimulation with KPC‐OVA cells (right graph) and subtracted of that evaluated in the presence of parental KPC cells. Data are represented as mean ± SD. *p ≤ .0332, **p ≤ .0021. (D) Degranulation activity was evaluated as CD107 positivity in CD8^+^ (left) and CD4^+^ (right) T cells isolated from spleens of immunised (filled pattern) and not (empty pattern) IL17A^+/+^ (black) and IL17A^−/−^ (blue) mice and exposed to either KPC‐OVA or parental KPC cells. Data are represented as mean fold change ± SD of CD107 positivity in the presence of KPC‐OVA cells, compared to parental KPC cells **p ≤ .0021, ***p ≤ .0002.

We previously demonstrated that IL17A‐deficient KPC mice exhibit increased CD8^+^ T‐cell infiltration and reduced regulatory T‐cell presence within the TME.^9^ To determine whether the absence of IL17A amplifies antigen‐specific immune responses, we used OVA as a model antigen. IL17A^+/+^ and IL17A^−/−^ C57BL/6 mice were immunised with mitomycin C‐treated KPC cells overexpressing OVA (mKPC‐OVA), followed by a challenge with live KPC‐OVA cells 3 weeks later (Figure 1A).

At the time of the boost (*post‐prime*), both non‐immunised IL17A^+/+^ and IL17A^−/−^ mice showed comparable levels of total anti‐OVA IgG. However, IL17A^−/−^ mice exhibited a significant skewing of IgG subclasses‐marked by reduced IgG1 (Th2‐associated) and increased IgG2c (Th1‐associated)—compared to IL17A^+/+^ mice. These differences became more pronounced post‐boost, where IL17A^−/−^ mice demonstrated a threefold increase in total anti‐OVA IgG, which was not observed in IL17A^+/+^ controls, even in non‐immunised animals. This suggests that IL17A may suppress the baseline humoral response and influence isotype switching independently of immunisation (Figure 1B).

Splenocytes from IL17A^−/−^ mice demonstrated a stronger cellular response, with increased numbers of IFN‐γ‐secreting cells upon OVA stimulation, particularly within the CD8^+^ T‐cell population (Figure 1C). CD8^+^ and CD4^+^ T cells from IL17A^−/−^ mice also expressed higher levels of CD107a, indicative of heightened cytotoxic degranulation, especially in response to KPC‐OVA compared to parental KPC cells (Figure 1D).

### 3.2 IL17A deficiency improves vaccine‐induced anti‐tumour immunity and survival

**FIGURE 2:**
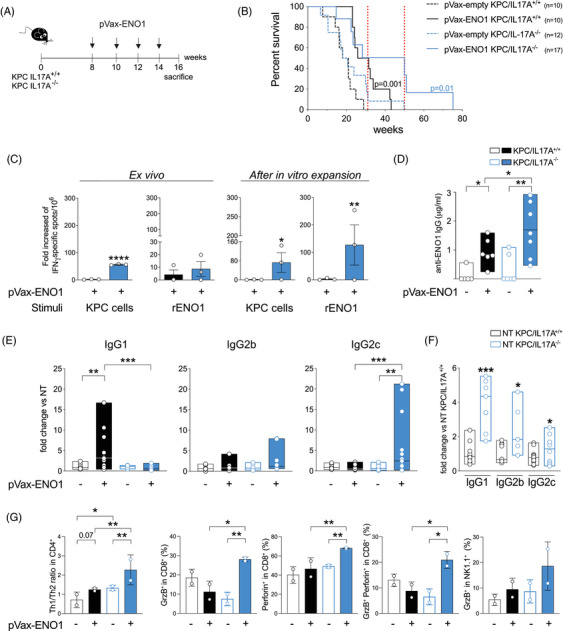
The absence of IL17A further increases DNA vaccine efficacy in prolonging pancreatic ductal adenocarcinoma (PDA)‐bearing mouse survival. (A) Treatment protocol of KPC/IL17A^+/+^ (*n* = 10) or KPC/IL17A^−/−^ (*n* = 12‐17) mice with ENO1 DNA vaccine. (B) Kaplan–Meier analysis of KPC/IL17A^+/+^ (black line) and KPC/IL17A^−/−^ (blue line) mice vaccinated with an empty (dotted line) or ENO1‐expressing (continuous line) pVax vectors. Red dotted lines indicate the time points in which 50% and 31% of ENO1 vaccinated KPC/IL17A^−/−^ mice were alive. (C) Splenocytes from pVax‐empty or pVax‐ENO1 vaccinated KPC/IL17A^+/+^ (black) and KPC/IL17A^−/−^ (blue) mice (*n* = 6) were assessed for the specific secretion of IFN‐γ in response to syngeneic KPC cells or rENO1, both ex vivo (left graphs) and after 1 week of in vitro stimulation in the presence of rENO1 (right graphs) as indicated. rENO1 was used only for in vitro stimulation and not injected into mice. Columns represent the average ± SD of the number of specific spots from vaccinated splenocytes divided by those from non‐vaccinated ones. Each dot represents a pool of two mice. *p < .05, **p < .005 and ****p < .0001 (D) Specific anti‐ENO1 antibodies were evaluated by a direct ELISA with sera from KPC/IL17A^+/+^ (black) and KPC/IL17A^−/−^ (blue) mice (*n* = 6‐8) vaccinated with pVax‐empty (empty pattern) or pVax‐ENO1 (filled pattern) vectors. Floating bars represent values from min to max ± SD; median is indicated. *p ≤ .0332, **p ≤ .002. (E) Anti‐ENO1 IgG1, IgG2b and IgG2c subclasses were evaluated by direct ELISA with sera from pVax‐empty (empty pattern) or pVax‐ENO1 vaccinated (filled pattern) KPC/IL17A^+/+^ (black) and KPC/IL17A^−/−^ (blue) mice. Data are represented as fold change of each serum over the mean value of the relative untreated control for both genotypes. Floating bars indicate the range from the minimum to the maximum ± SD; median is shown. Each dot represents technical replicates of *n* = 3–6 sera. **p ≤ .0021, ***p ≤ .0002. (F) Anti‐ENO1 IgG1, IgG2b, and IgG2c subclasses in the sera from untreated mice, expressed as fold change of each serum over the mean value of sera from the untreated KPC/IL17A^+/+^ mice. Floating bars indicate the range from the minimum to the maximum ± SD; median is shown. Each dot represents technical replicates of *n* = 3–6 sera. *p ≤ .0332, ***p ≤ .0002 indicate significant differences between KPC/IL17A^−/−^ (blue) and KPC/IL17A^+/+^ (black) sera. (G) Flow cytometry analysis of splenocytes from pVax‐empty (empty pattern) or pVax‐ENO1 vaccinated (filled pattern) KPC/IL17A^+/+^ (black) and KPC/IL17A^−/−^ (blue) mice (n = 4‐5). Bars represent the average ± SD of the ratio or percentage of positive cells stained for markers indicated in the y‐axis. Each dot represents a pool of 2–3 biological replicates. Outliers are removed after running ROUT analysis. *p ≤ .0332, **p ≤ .0021.

To assess whether IL17A depletion augments vaccine‐induced tumour immunity, KPC/IL17A^+/+^ and KPC/IL17A^−/−^ mice were vaccinated with ENO1‐pVAX starting at 2 months of age (Figure 2A).^14^ ENO1 vaccine significantly extended survival in both genotypes. However, IL17A^−/−^ mice demonstrated superior long‐term survival, with 50% of vaccinated mice alive at 32 weeks, compared to only 30% of IL17A^+/+^ counterparts. At 50 weeks, 31% of vaccinated IL17A^−/−^ mice remained alive, while all IL17A^+/+^ mice had succumbed (Figure 2B). The percentages obtained from the log‐rank test (# at risk) are reported here for clarity and consistency with Figure 2B.

Vaccinated IL17A^−/−^ mice exhibited a significantly enhanced T‐cell response to recombinant ENO1 and syngeneic KPC cells, both ex vivo and after in vitro stimulation (Figure 2C). The presence of IFN‐γ spots in response to MHC class I‐expressing KPC cells confirmed robust CD8^+^ T‐cell activation. IL17A deficiency also elevated anti‐ENO1 antibody production and skewed the IgG subclass profile towards Th1‐associated IgG2c while reducing IgG1 (Figure 2D,E). Notably, untreated IL17A^−/−^ mice also produced higher baseline levels of all IgG subclasses, compared to IL17A^+/+^ mice (Figure 2F).

Flow cytometry analysis of IFN‐γ and IL4 production by splenocytes from vaccinated and non‐vaccinated KPC/IL17A^+/+^ and KPC/IL17A^−/−^ mice revealed a significant increase in Th1 over Th2 cells in the absence of IL17A (Figure 2G). This Th1 polarisation was accompanied by a greater proportion of CD8^+^ T cells expressing granzyme B and perforin, either individually or co‐expressed, indicating enhanced cytotoxic potential. Additionally, NK1.1^+^ cells from KPC/IL17A^−/−^ mice showed elevated granzyme B expression, although to a lesser extent than CD8^+^ T cells (Figures 2G and S1A,B).

### 3.3 IL17A absence alters tumour immune infiltration in response to cancer vaccination

**FIGURE 3:**
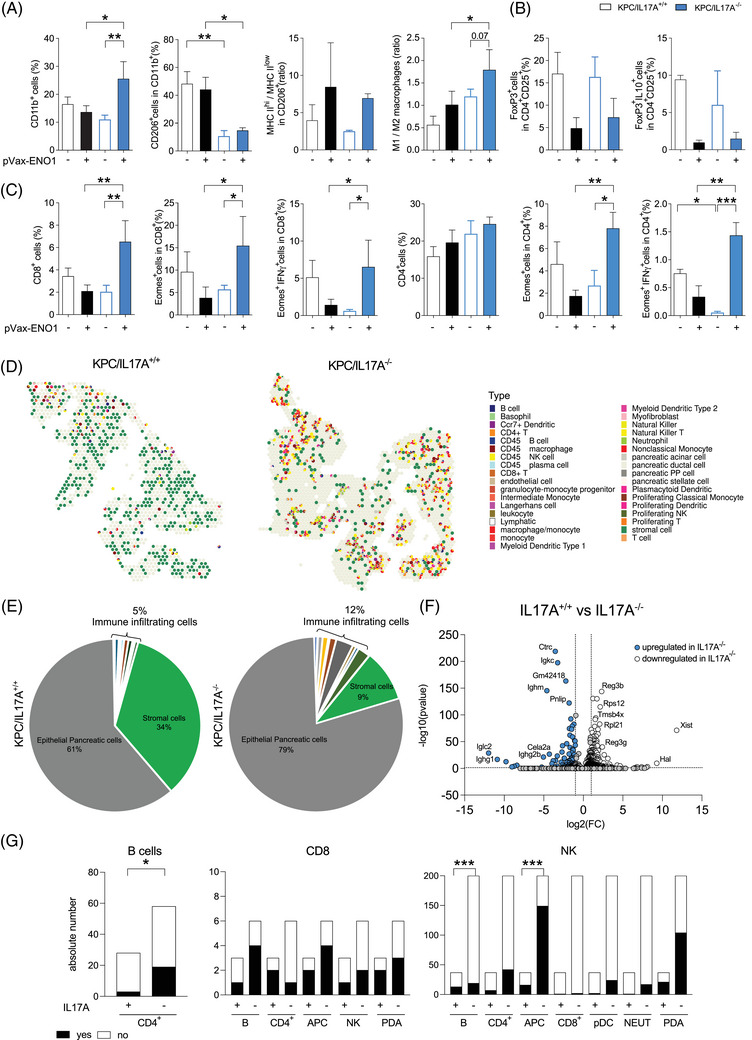
The absence of IL17A strongly impacts the heterogeneous immune landscape induced by the ENO1‐DNA vaccine. (A–C) Percentage of PDA‐infiltrating myeloid cells (A), regulatory T cells (B) and effector/memory CD4^+^ and CD8^+^ T cells (C) in tumours from ENO1‐vaccinated (filled columns) or not (empty columns) KPC/IL17A^+/+^ (black columns) or KPC/IL17A^−/−^ (blue columns) mice (*n* = 3–6) evaluated through flow cytometry. Markers on the y‐axis title define the different subpopulations. Columns represent the average number ± SEM. *p ≤ .0332, **p ≤ .0021, ***p ≤ .0002. (D) Spatial transcriptomic analysis of OCT‐embedded pancreatic tissues from ENO1‐vaccinated KPC/IL17A^+/+^ (left) and KPC/IL17A^−/−^ (right) mice: cell clusters are depicted on the tissue samples; colors indicate different cell types as reported in the legend. (E) Pie charts showing percentages of cell subtypes in vaccinated KPC/IL17A^+/+^ (left) and KPC/IL17A^−/−^ (right) tissues. (F) Differentially expressed genes are depicted in the volcano plot. Blue dots represent transcripts increased and white dots those decreased in IL17A^−/−^ tumours. (G) Quantification of B cells (left), CD8^+^ T cells (middle), and NK cells (right) that are either co‐localised (black) or not co‐localised (white) with the x‐axis–indicated cell type, based on cell counts per spot (as shown in panel D), in tumours from ENO1‐vaccinated KPC mice that are IL17A‐proficient (+) or ‐deficient (–). *p < .05 and ***p < .0001.

Flow cytometry of tumours from ENO1‐vaccinated mice revealed a distinct immune infiltrate profile in KPC/IL17A^−/−^ mice. These tumours contained more total CD45^+^ and CD11b^+^ myeloid cells with fewer CD206^+^ M2‐like macrophages and more M1‐like cells as noted by the M1/M2 ratio (Figures 3A and S1A,C).^19^ The vaccine reduced Treg and IL10‐secreting T cells in both genotypes but induced a significantly higher influx of CD8^+^, and CD4^+^ T cells to a lesser extent, in IL17A^−/−^ mice (Figure 3B). Effector/memory T cells expressing Eomes transcriptional factor^20^ and producing IFN‐γ were markedly increased in this group (Figure 3C).

To spatially contextualise the immune infiltrates identified by flow cytometry, an exploratory spatial transcriptomics analysis was conducted on tumours from ENO1‐vaccinated KPC/IL17A^+/+^ and IL17A^−/−^ mice (Figures 3D–G and S2). Tissues were processed with H&E (Supporting Information Figure S2A). Spots representing modulated genes were captured by 10X Genomics Visium, clustered in 33 clusters and aligned to H&E images (Figure 3D). First, spatial transcriptomic analysis confirmed the above findings. ENO1‐vaccinated IL17A^−/−^tumours displayed a more than twofold increase in immune cell infiltration, including CD4^+^, CD8^+^ T and NK cells, which preferentially co‐localised with antigen‐presenting cells, and B cells (Figures 3D,E,G and S2B,C). Among the top genes differentially expressed in the presence of vaccination but the absence of IL17A, there are immunoglobulin genes (Figure 3F). This along with a greater CD4^+^ T‐cell interaction with B cells in IL17A^−/−^ tumours (Figure 3G) suggests an enhanced humoral response and B‐cell activation. Although stromal cell abundance was higher in IL17A^+/+^ tumours, gene expression patterns among inflammatory (iCAF), myofibroblastic (myCAF) and antigen‐presenting (apCAF) subtypes were similar between groups (data not shown).

### 3.4 IL17A depletion unleashes cytotoxic T cells against tumour cells

**FIGURE 4:**
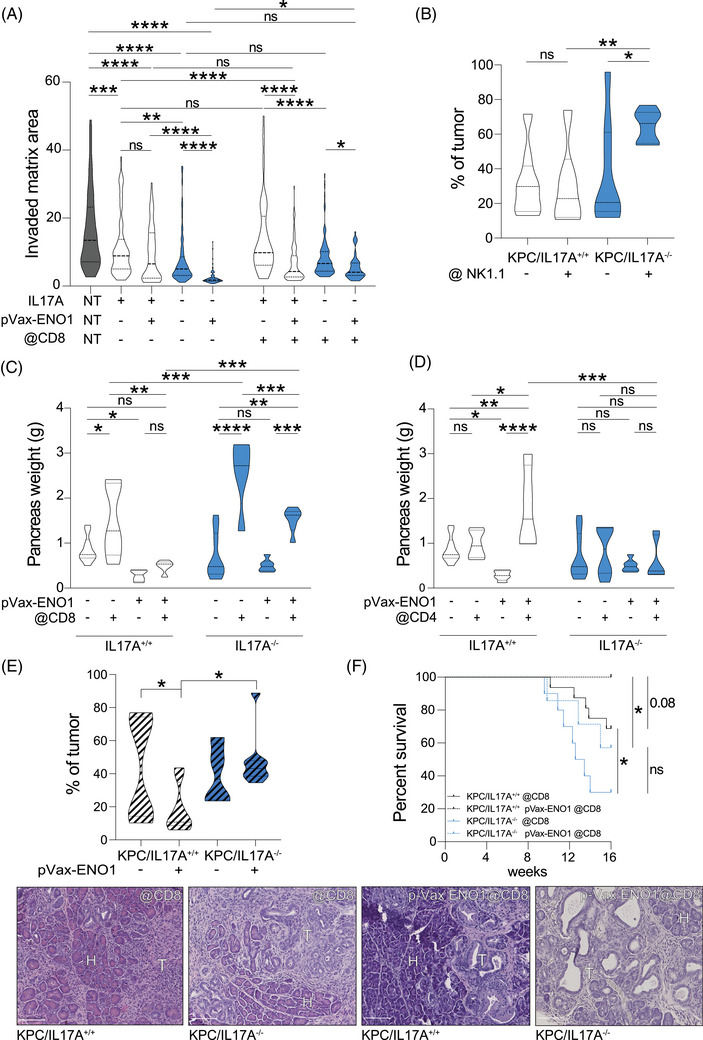
IL17A absence allows the activation of CD8^+^ T cells by an anti‐cancer vaccine. (A) Splenocytes from pVax‐ENO1 vaccinated or not KPC/IL17A^+/+^ (white) or KPC/IL17A^−/−^ (blue) mice (*n* = 3) were co‐cultured with spheroids generated with syngeneic KPC cells for 48 h. Truncated violin plots represent measurement of spheroid invasion of the surrounding matrix at Day2 vs. Day0. The right part of the graph reports data obtained with splenocytes from mice depleted of CD8^+^ T cells. *p ≤ .0332, **p ≤ .0021, ***p ≤ .0002, ****p < .0001. Not‐significant differences (ns) are reported. (B) Truncated violin plots show the percentage of tumour in KPC/IL17A^+/+^ (white) or KPC/IL17A^−/−^ (blue) mice (*n* = 6–8) treated or not with an anti‐NK1.1 antibody. *p ≤ .0332, **p ≤ .0021. Not‐significant differences (ns) are reported. (C) Truncated violin plots show the tumour weight of C57BL/6 IL17A^+/+^ (white) and IL17A^−/−^ (blue) mice (*n* = 7–9) vaccinated or not with pVax‐ENO1 and depleted of CD8^+^ T cells. *p ≤ .0332, **p ≤ .0021, ***p ≤ .0002, ****p < .0001. Not‐significant differences (ns) are reported. (D) Truncated violin plots show the tumour weight of C57BL/6 IL17A^+/+^ (white) and KPC/IL17A^−/−^ (blue) mice (*n* = 5–9) vaccinated or not with pVax‐ENO1 and depleted or not of CD4^+^ T cells. *p ≤ .0332, **p ≤ .0021, ***p ≤ .0002, ****p < .0001. Not‐significant differences (ns) are reported. (E) Truncated violin plots show the percentage of tumour in KPC/IL17A^+/+^ (white) or KPC/IL17A^−/−^ (blue) mice (*n* = 8–10) depleted of CD8^+^ T cells (dashed pattern) and vaccinated or not with pVax‐ENO1. *p ≤ .0332 (f) Kaplan–Meier analysis of KPC/IL17A^+/+^ (black lines) and KPC/IL17A^−/−^ (blue lines) mice depleted of CD8^+^ T cells and vaccinated with pVax‐ENO1 (dotted lines) or not (continuous lines). Representative H&E‐stained pancreatic sections are shown with tumour (T) and healthy (H) areas indicated. Scale bar is 100 µm.

To assess cytotoxic function, splenocytes from vaccinated and unvaccinated mice (Figure 2A) were co‐cultured with KPC‐derived tumour spheroids. Splenocytes from KPC/IL17A^−/−^ mice exhibited significantly greater tumoricidal activity, with ENO1‐vaccinated IL17A^−/−^ splenocytes reducing spheroid growth by 86%, compared to 50% in vaccinated IL17A^+/+^ mice (Figures 4A and S3A,B). CD8^+^ T‐cell depletion impaired this response only in the IL17A^−/−^ group, underscoring the pivotal role of CD8^+^ T cells. Residual killing activity following CD8^+^ T‐cell depletion in IL17A^−/−^ splenocytes suggested a contributory role of NK cells, which was confirmed by increased tumour growth following NK1.1 cell depletion in IL17A^−/−^ mice, as shown in the representative H&E images (Figures 4B and S3D).

Orthotopic injection of KPC cells into CD8^+^ T cell‐depleted IL17A^−/−^ and IL17A^+/+^ mice further validated these findings (Figures 4C and S3D). CD8^+^ T‐cell absence negated the therapeutic effect of ENO1 vaccination in IL17A^−/−^ mice, while CD4^+^ T cells were more critical for vaccine efficacy in IL17A^+/+^ mice (Figure 4D). Overall survival analysis reinforced the essential role of CD8^+^ T cells in mediating tumour control in the IL17A‐deficient setting (Supporting Information Figure S3E). CD8⁺ T‐cell depletion increased tumour burden in both IL17A‐proficient and ‐deficient KPC mice (data not shown). However, this effect was markedly more pronounced in ENO1‐vaccinated KPC/IL17A^−/−^ mice, which showed significantly increased tumour area and reduced survival, compared to KPC/IL17A^+/+^ controls (Figure 4E,F), highlighting the pivotal role of CD8⁺ T cells in mediating tumour control in the absence of IL17A.

### 3.5 Pharmacological IL17A blockade recapitulates genetic depletion outcomes

**FIGURE 5:**
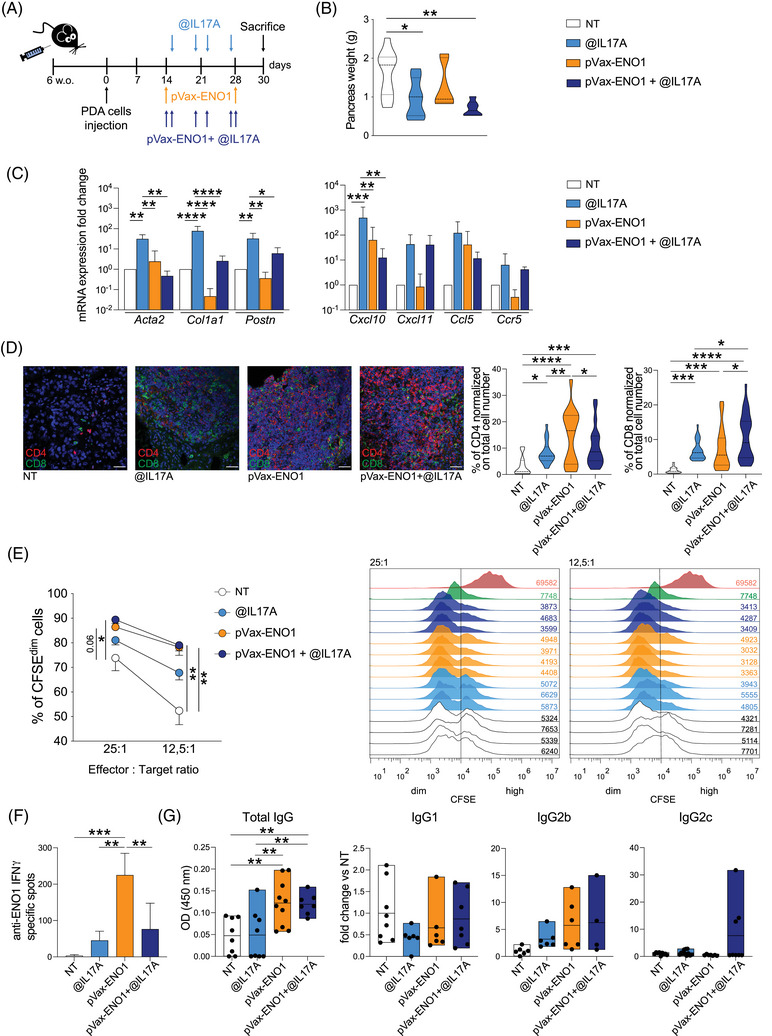
Orthotopic model confirms the effects of the IL17A pharmacological depletion. (A) Scheme of combined therapy in mice injected orthotopically with syngeneic KPC cells. (B) Pancreas weight was evaluated from mice receiving the different treatments (*n *= 8–12). Truncated violins represent min to max values ± SD. *p ≤ .0332, **p ≤ .0021. (C) Stromal‐ (left graph) and T‐cell activation‐ (right graph) related transcripts were evaluated by Quantitative real‐time polymerase chain reaction (qRT‐PCR) from formalin‐fixed and paraffin‐embedded tissues (*n* = 4–6). Columns represent mean ± SD. *p ≤ .0332, **p ≤ .0021, ***p ≤ .0002, ****p < .0001. (D) Representative immunofluorescence pictures of CD4^+^ and CD8^+^ T cells infiltrating tumours from mice receiving the indicated treatments (*n* = 4–5). Truncated violin plots represent quantification of CD4^+^ (left) and CD8^+^ (right) T cells in five representative fields for each mouse. *p ≤ .0332, **p ≤ .0021, ***p ≤ .0002, ****p < .0001. Scale bar is 50 µm. (E) Percentage of PDA CFSE^dim^ cells (left graph) 4 h after their co‐culture with splenocytes (25:1 and 12,5:1 E:T ratios) from mice receiving the indicated treatments (*n* = 3–4). The overlaid histograms (right graphs) represent the PDA CFSE^hi^ (right of the line) and CSFE^dim^ (left of the line) in the positive (pink peak) and negative (green peak) controls and in cells co‐cultured with splenocytes from individual mice receiving the different treatments. Data from the two different E:T ratios are represented, with a geometric mean of fluorescence indicated in the histograms. *p ≤ .0332, **p ≤ .0021. One representative of two independent experiments is shown. (F) IFN‐γ‐secreting splenocytes isolated from mice of each treatment group (*n* = 5) were stimulated in the presence or absence of rENO1 in Enzyme‐Linked ImmunoSPOT (ELISpot) plates. Columns represent the average number ± SD of specific spots evaluated in the presence of rENO1 and subtracted of those evaluated when splenocytes were cultured in medium only. **p ≤ .0021, ***p ≤ .0002. (G) Anti‐ENO1 total IgG, IgG1, IgG2b and IgG2c subclasses were measured through a direct ELISA with sera from mice receiving the different treatments. For IgG1, IgG2b and IgG2c subclasses data are represented as fold change of each serum over the mean value of the untreated serum group. Floating bars represent min to max values ± SD; median is indicated. Each circle represents technical replicates of mouse sera (*n* = 5). Outliers are removed after running ROUT analysis. **p ≤ .0021.

To translate these findings into a clinically relevant model, we treated orthotopically implanted KPC‐bearing mice with anti‐IL17A mAb, with or without ENO1 vaccination (Figure 5A). While monotherapies moderately reduced tumour burden, combination treatment significantly decreased tumour weight (Figure 5B).

Gene expression analysis revealed that anti‐IL17A mAb induced changes in stromal (e.g., Collagen I, α‐Smooth Muscle Actin (SMA),^21^ Periostin^22^) and immune‐related genes (e.g., CXCL10,^23^, ^24^ CXCL11,^25^ CCL5, CCR5^26^), mirroring the KPC/IL17A^−/−^ model^9^ (Figure 5C). Immunofluorescence confirmed increased T‐cell infiltration in all groups, with CD8^+^ T‐cell recruitment most prominent in the combination group and CD4^+^ T cells in the vaccination group (Figure 5D). Splenocytes from the combination group showed a modest increase in cytotoxicity and IFN‐γ secretion, compared to monotherapy groups (Figure 5E,F), although the effect was less pronounced than in the spontaneous model (Figure 4A) due to the different treatment schedule (two vs. four rounds of vaccination) and the time of sacrifice. Analysis of antibody responses revealed comparable levels of total anti‐ENO1 IgG between the combination and vaccine‐only groups following outlier correction, but a trend towards increased IgG2c levels was retained (Figure 5G), suggesting a possible shift towards a Th1‐associated profile. These results suggest that pharmacological IL17A blockade can enhance immune activation and contribute to the anti‐tumour effect of the ENO1 DNA vaccine, even in a rapidly progressing orthotopic model where the vaccine alone shows limited efficacy.

## 4 DISCUSSION

In this study, we aimed to demonstrate that IL17A depletion represents a new approach to increase the intensity of an antigen‐specific cytotoxic and humoral response and, therefore, the effectiveness of a cancer vaccine applied in the treatment of PDA (Supporting Information Figure S4). Cancer vaccines re‐emerge again as promising immunotherapeutic approaches,^27^ even in pancreatic cancer.^28^, ^29^ Here, the TME exerts profound influence on tumour progression, therapeutic response and clinical outcomes through intricate signalling cascades and molecular interactions.^30^ However, the use of a personalised mRNA vaccine coding for 20 specific neoantigens (mutated proteins specifically expressed by tumour cells due to sporadic somatic gene mutations) was able to significantly induce a specific T‐cell response in 50% of vaccinated patients and prolong the period of disease‐free survival after surgery.^31^ Given that IL17A‐targeted monoclonal antibodies (e.g., secukinumab) are already FDA‐approved for autoimmune diseases with well‐characterised safety profiles, combining such agents with neoantigen or ENO1 vaccines could be rapidly tested in early‐phase PDA trials. Patient selection could be guided by baseline IL17A levels in serum or tumour, enabling a precision‐medicine approach. Our vaccine approach was focused on a widely expressed antigen, crucial for tumour cell progression, and for which most patients present a spontaneous immune response. This antigen is ENO1, previously defined as a moonlight protein due to its multiple locations and functions in supporting tumour metabolism, growth, survival and invasiveness.^31^, ^32^, ^33^ More than 60% of PDA patients displayed anti‐ENO1 antibodies in their sera, especially against two more acidic isoforms with phosphorylated residues,^34^ and two‐thirds of patients with anti‐ENO1 Ab also had T cells secreting IFN‐γ in response to recombinant ENO1.^35^ Notably, the antibody response to ENO1 increased in patients who received two cycles of chemotherapy,^15^ highlighting the possibility that the ENO1 vaccine may boost the anti‐tumoural response of those PDA patients who cannot be surgically resected and receive chemotherapy alone. ENO1‐DNA vaccine significantly prolonged survival in the KPC mouse model by eliciting an integrated humoral and cellular immune response,^13^, ^36^ which was amplified by the combination with gemcitabine.^15^ While the vaccine delayed tumour progression, immunosuppressive cells and tumour eventually arose again.^14^ Moreover, the ENO1‐DNA vaccine mainly elicited CD4^+^ T cells, compared to the mRNA neoantigen vaccine, which induced CD8+ T cells only.^28^ Therefore, the idea of combining some strategy with the DNA vaccine to also recruit and activate CD8^+^ T cells was considered. When we observed that KPC/IL17A^−/−^ tumours were much more infiltrated by CD8^+^ T cells and significantly less by Treg,^9^ we thought to exploit the TME reshaping mediated by the IL17A's depletion to improve the ENO1‐DNA vaccine efficacy.

IL17A directly affects KPC cells, macrophages, neutrophils,^12^, ^37^, ^38^ all of which express its receptors and maintains immunosuppression and immune checkpoint inhibitor resistance. IL17A produced by CD8^+^ T cells (called Tc17) synergises with tumor necrosis factor (TNF) in inducing differentiation of iCAFs. These cells, in turn, secrete IL6, which maintains the transforming growth factor (TGF)β‐dependent differentiation of Tc17 in a positive loop.^39^ Finally, the presence of IL17A can also be stimulated by fungal overgrowth, which has been linked to a reduced immune response in breast cancer and melanoma.^40^ In PDA, the presence of fungi is associated with both the activation of the complement cascade via mannose‐binding lectin and the induction of a Th2‐type response following IL33 secretion.^41^, ^42^

In this study, we vaccinated KPC/IL17A^−/−^ mice with the ENO1‐DNA vaccine^14^ and observed differences in longer survivors (KPC/IL17A^−/−^ mice only survived longer than 42 weeks of age) and a significant improvement in the anti‐tumoural response. Indeed, spatial transcriptomic analysis confirmed the hypothesis that the absence of IL17A modifies the TME by licensing the stroma to recruit immune cells, a process further promoted by DNA vaccination. Importantly, human PDA single‐cell RNA‐seq datasets similarly identify IL17A‐expressing T‐cell clusters in the stroma that correlate with poorer survival.^10^, ^39^ This parallel suggests our murine TME remodelling findings may translate directly into the human disease setting. Myeloid cells recruited in the absence of IL17A seem to function as antigen‐presenting cells, resulting in higher MHC II expression, while T cells exhibit a more effector/memory profile. An abundance of transcripts related to NK cells and cytotoxic activity was observed in vaccinated KPC/IL17A^−/−^ mice, more than in vaccinated KPC/IL17A^+/+^ mice. While the spatial transcriptomics analysis was conducted with a limited sample size and primarily for exploratory purposes, the findings reinforced our phenotypic and functional data, particularly regarding immune cell infiltration and interaction within the TME. NK cells, as well as neutrophils, are more abundant in tumours in the absence of IL17A, but especially the huge antibody production induced by the ENO1 vaccine could be responsible for the antibody‐dependent cytotoxicity against tumour cells. This was confirmed by the intradot neighbourhood analysis that highlighted the spatial connection of NK, antigen‐presenting cells, plasmacytoid dendritic cells (DC) secreting IFN and pancreatic cells. Furthermore, CD8^+^ T cells were significantly more abundant both in vaccinated KPC/IL17A^−/−^ mice and in the orthotopic tumours treated with an anti‐IL17A mAb. Depletion of these cells strongly impaired the effect of the ENO1‐DNA vaccine in limiting tumour growth and improving survival in IL17A‐deficient mice, while in IL17A^+/+^ mice, the role of CD4^+^ T cells was much more crucial. A study conducted on 57 patients demonstrated that high infiltration of CD3^+^ TILs and high collagen density correlated significantly with better overall survival and progression‐free survival.^43^ Similarly, high tumour infiltration of CD3^+^ and CD8^+^ T cells was related to a better overall and progression‐free survival in the Kaplan–Meier analysis. Furthermore, the group of patients exhibiting CD3‐, CD8‐ or CD20‐positive cells demonstrated a significant correlation with improved survival at 24 months following primary tumour resection.^43^ Conversely, in *Trypanosoma cruzi* infection, IL17RA‐deficient CD8^+^ T cells showed dysfunction, suggesting a role for IL17A and F in sustaining survival and preventing apoptosis of pathogen‐specific CD8^+^ T cells.^44^ It is noteworthy that in KPC/IL17A^−/−^ mice, NK cells depletion significantly restarted tumour growth but did not affect tumours in KPC/IL17A^+/+^ mice. Interestingly, NK cell depletion in KPC/IL17A^+/+^ mice did not result in worsened tumour progression and appeared to be associated with more preserved tissue morphology as shown by H&E staining. While this effect was not statistically significant, it supports the idea that NK cells may play a limited role in tumour control in IL17A‐proficient contexts, in line with our previous findings that CD4⁺ T cells are the predominant effectors in these settings.^15^ This may be related to the intense humoral response observed in the absence of IL17A: Indeed, an increase in total specific anti‐ENO1 IgG and, in particular, those subclasses induced by a Th1 response and IFN‐γ was evaluated. In contrast, those more associated with a Th2 response (e.g., IL4 and IL5)^45^ were significantly reduced in the absence of IL17A. Furthermore, the absence of IL17A led to a continuous increase in the production of anti‐OVA IgG rather than in the presence of IL17A, where probably a sort of B cell anergy occurred. While the role of B cells in tumour immunity remains complex, our findings suggest that IL17A depletion may shift the immune environment to favour B‐cell activation and antibody production, possibly through reduced immunosuppressive cytokine signalling or altered antigen presentation within the tumour stroma. This is further supported by our spatial transcriptomics data, which revealed increased expression of immunoglobulin‐related genes in IL17A‐deficient, vaccinated tumours consistent with enhanced local B‐cell activity. Future studies will aim to directly visualise Tertiary Lymphoid Structure (TLS)‐like structures and further characterise B‐cell subsets in IL17A‐deficient settings. In PDA, the loss of all circulating antibodies as well as the loss of the activation‐induced cytidine deaminase, which allows the isotype switch of antibodies, similarly accelerated tumour progression. Of note, the lack of all antibodies led to an increased number of mice with lung and liver metastases and to an unexpected decrease in podoplanin^+^ CAFs and a reduction of extracellular matrix density.^46^ B cells retained within tertiary lymphoid structures or similar are associated with a favourable prognosis, whereas scattered or interspersed B cells within the stroma do not correlate with prolonged survival.^47^ B regulatory cells are indeed present in PDA, and the use of an inhibitor of the Bruton tyrosine kinase, which promotes the differentiation of regulatory B cells, has displayed promising results in terms of PanIN lesions reduction and of IFN‐γ‐CD8^+^ T cells influx into the tumour. IL10 and IL35 produced by regulatory B cells seem to be the two cytokines responsible for tumour proliferation and T‐cell suppression.^48^ Similarly, a phosphoinositide 3‐kinase gamma inhibitor was demonstrated to increase B cell and follicular T helper cell activation with a strong production of antibodies that mediate antibody‐dependent cytotoxicity.^49^

Overall, this study provides new insights into the effects of IL17A in modulating the intensity of both antigen‐specific humoral and cellular responses and the anti‐tumour response induced by a DNA anti‐cancer vaccine in pancreatic cancer. However, we speculate that the ability of this cytokine in remodelling the stroma and improving the immune response may also be effective in other tumours. Further investigation is needed, but the implication to translate the use of an anti‐IL17A into clinical practice to enhance immunotherapy response is of great relevance.

## AUTHOR CONTRIBUTIONS

Giorgia Tiberi, Alessandro Scagliotti, Claudia Curcio, Ermes Candiello, Francesco Novelli and Paola Cappello conceived the research. Giorgia Tiberi, Alessandro Scagliotti, Claudia Curcio, Ermes Candiello, Paula Ariadna Diez Villegas, Silvia Brugiapaglia, Gianluca Mucciolo, Cecilia Roux, Maddalena Arigoni, Giuseppina Barutello and Paola Cappello performed the experiments. Roberta Curto, Alessandro Scagliotti and Giorgia Tiberi cared the animal breeding and in vivo procedures. Raffaele A. Calogero and Mauro Giulio Papotti provided reagents. Giorgia Tiberi, Alessandro Scagliotti, Claudia Curcio, Ermes Candiello, Giancarlo Castellano, Niccolò Bolli and Paola Cappello analysed data and results. Giulia Adriani provided resource and technical insights for tumour spheroids and intellectual contribution to the discussion of data. Paola Cappello wrote the paper. Giorgia Tiberi, Alessandro Scagliotti, Claudia Curcio, Cecilia Roux, Mauro Giulio Papotti and Francesco Novelli revised the draft.

## ETHICS STATEMENT

All animal experiments were conducted in accordance with institutional guidelines, the European Union Directive 2010/63/EU, and the Italian Ministry of Health regulations. Mice were maintained under specific pathogen‐free (SPF) conditions at the animal facilities of the Molecular Biotechnology Centre “Guido Tarone.” Experimental protocols were approved by the Italian Ministry of Health (approval numbers: 597/2019‐PR and 861/2024‐PR). To minimize bias, only a limited number of researchers oversaw animal treatments. The study was designed and reported in compliance with the ARRIVE (Animal Research: Reporting of In Vivo Experiments) guidelines.

## Supporting information



Supporting Information

## Data Availability

All data are available upon inquiry to the corresponding author.
