# Supplementary material for: The absence of IL17A favours cytotoxic cell function and improves antigen‐specific immunotherapies in pancreatic adenocarcinoma
Source: Clin Transl Med. 2025 Aug 19;15(8):e70442. doi: 10.1002/ctm2.70442 (PMC12364998; doi:10.1002/ctm2.70442)
Supplement: Supplementary file 1 — Supporting Information [file CTM2-15-e70442-s001.pdf]

Supplementary Figure 1

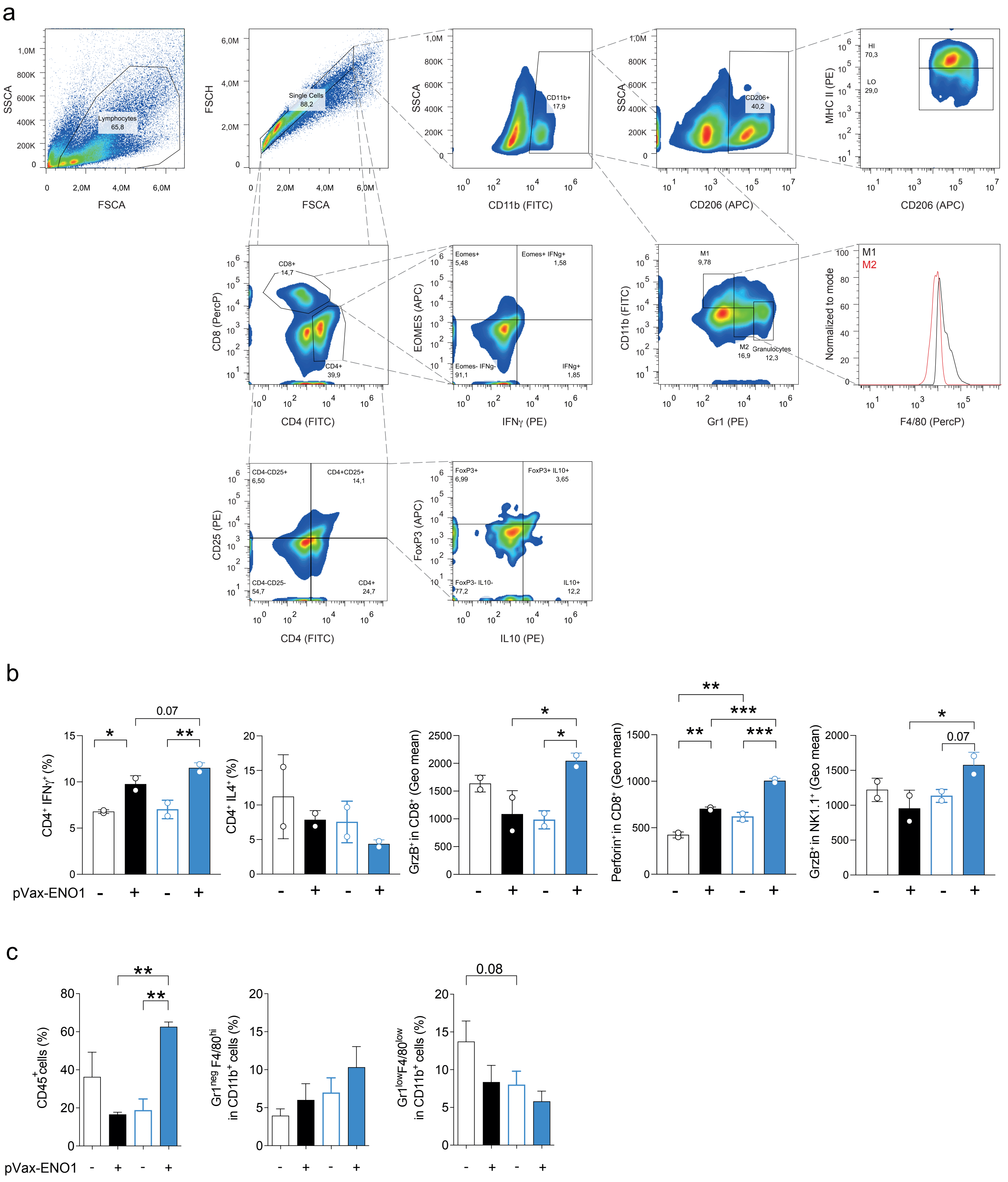

Supplementary Fig 1. Gating strategy of PDA-infiltrating immune cells. (a) The infiltrating immune populations and singlets were selected based on physical parameters (first and second panel). Dotted lines indicate the gating strategy performed for myeloid subpopulation, effector and regulatory T cells. (b) Flow cytometry evaluation of the percentage of IFNγ and IL4 by CD4+ T cells, and the geometric mean of Granzyme and Perforin produced by CD8+ and NK1.1+ cells from pVax-empty (empty pattern) or pVax-ENO1 vaccinated (filled pattern) KPC/IL17A+/+ (black) and KPC/IL17A-/- (blue) splenocytes.Bars represent the average ± SD. Each dot represents a pool of 2-3 biological replicates. (c) Percentage of tumor infiltrating cells positive for the y axis-indicated markers from pVax-empty (empty pattern) or pVax-ENO1 vaccinated (filled pattern) KPC/IL17A+/+ (black) and KPC/IL17A-/- (blue) splenocytes (n=3-6). Bars represent the average ± SEM. *≤ 0.0332, **≤ 0.0021, ***≤ 0.0002.

Supplementary Figure 2

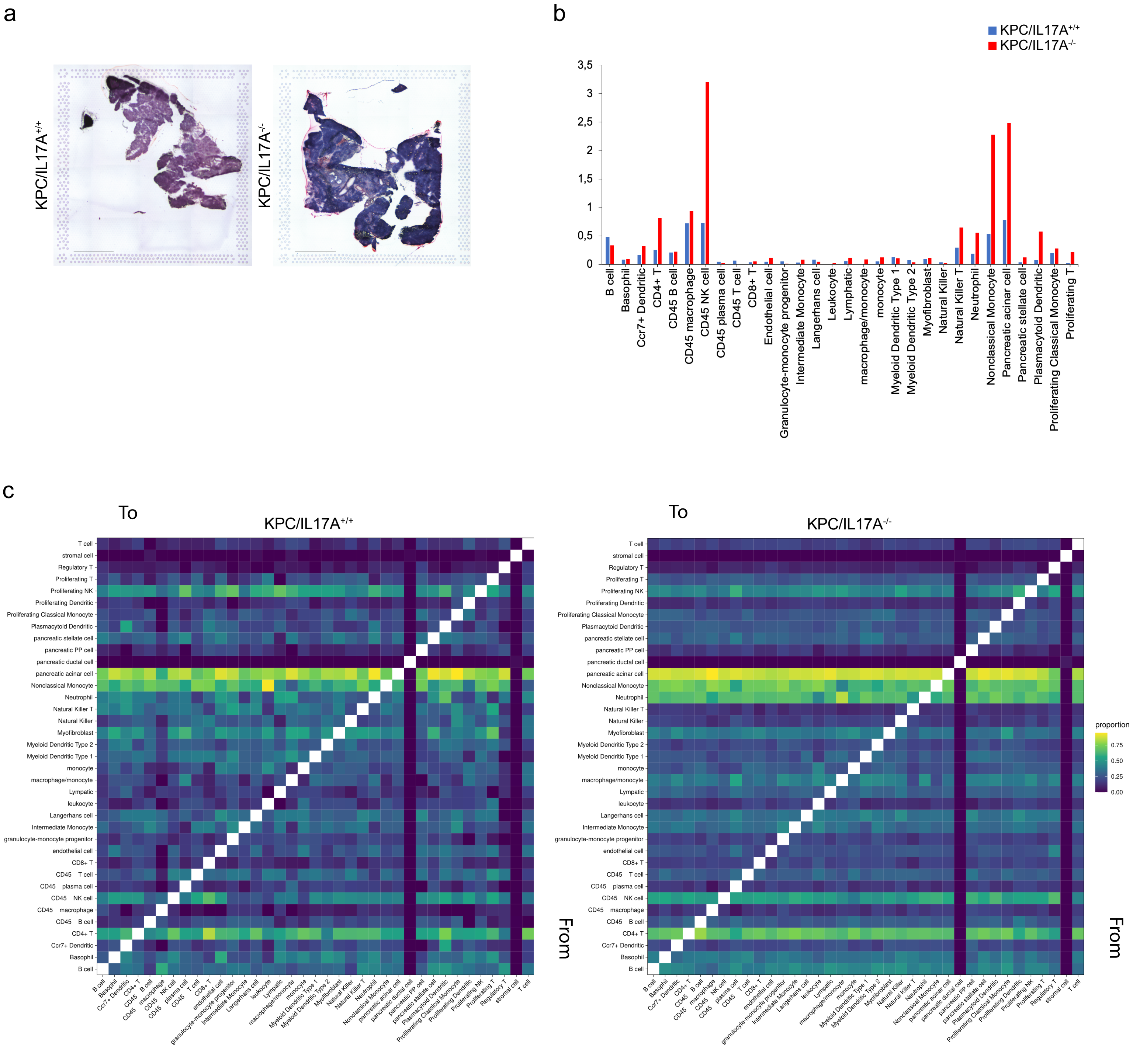

Supplementary Fig 2. Spatial transcriptomic analysis of OCT-embedded pancreatic tissues from KPC/IL17A$^{+/+}$ and KPC/IL17A$^{-/-}$ mice. (a) Haematoxylin and Eosin staining of sections processed with Visium^{TM} technology. (b) Percentage of immune infiltrating cells was evaluated in ENO1-vaccinated KPC/IL17A$^{+/+}$ (blue) and KPC/IL17A$^{-/-}$ (red) mice. (c) Intradot neighborood analysis in ENO1-vaccinated KPC/IL17A$^{+/+}$ (left) and KPC/IL17A$^{-/-}$ (right) mice is represented as heatmap; colors indicate score proportion of interaction among different cell types.

Supplementary Figure 3

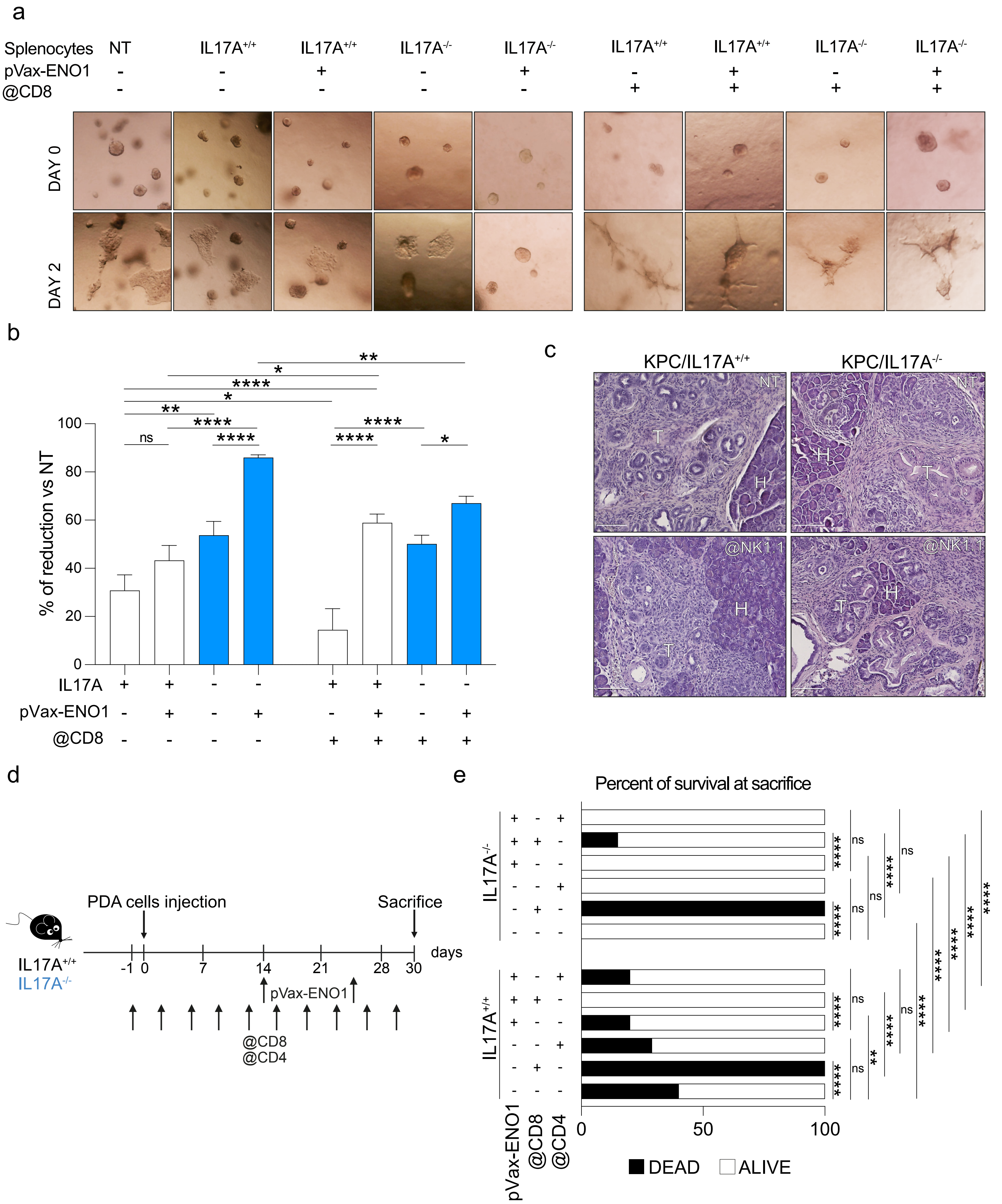

Supplementary Fig 3. (a) Representative images at day 0 (upper raw) and day 2 (lower raw) of spheroids obtained from KPC cells challenged in a 3D matrix and co-cultured with splenocytes from ENO1-vaccinated or not KPC/IL17A$^{+/+}$ or KPC/IL17A$^{-/-}$ mice and *in vivo* depleted or not of CD8^{+} T cells (n=3). (b) Histograms show the percentage of reduced area of spheroids exposed to splenocytes from ENO1-vaccinated or not KPC/IL17A$^{+/+}$ (white columns) or KPC/IL17A$^{-/-}$ (blue columns) mice and *in vivo* depleted or not of CD8^{+} T cells. *p ≤ 0.0332, **p ≤ 0.0021, ****p < 0.0001. Not-significant differences (ns) are reported. Data are represented as the percentage of reduction compared to the growth of untreated spheroids. (c) Representative H&E-stained pancreatic sections of KPC/IL17A$^{+/+}$ or KPC/IL17A$^{-/-}$ depleted or not of NK1.1. Tumor (T) and healthy (H) areas are indicated. Scale bar is 100 μm. (d) Treatment protocol of mice orthotopically injected with singeneic KPC cells, vaccinated or not with pVax-ENO1 and depleted or not of CD8^{+} or CD4^{+} T cells. (e) Bars indicate percentage of mice of the orthotopic experiment alive (white) and dead (black) at time of sacrifice (30 days). **p < 0.005 and ****p < 0.0001. Not-significant differences (ns) are reported.

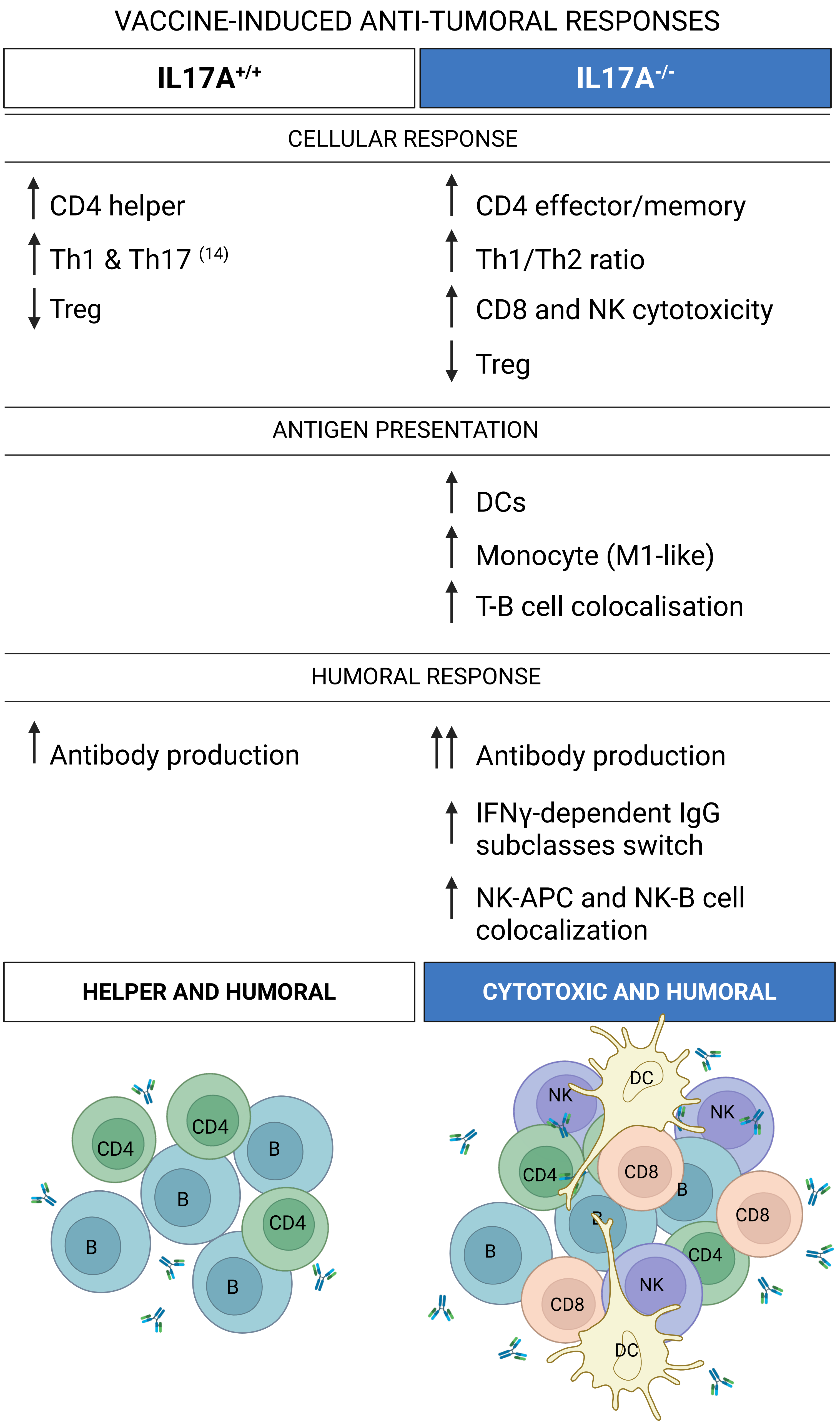
VACCINE-INDUCED ANTI-TUMORAL RESPONSES
IL17A+/+
IL17A-/-
CELLULAR RESPONSE
CD4 helper
Th1 & Th17 (14)
Treg
CD4 effector/memory
Th1/Th2 ratio
CD8 and NK cytotoxicity
Treg
ANTIGEN PRESENTATION
DCs
Monocyte (M1-like)
T-B cell colocalisation
HUMORAL RESPONSE
Antibody production
Antibody production
IFNγ-dependent IgG subclasses switch
NK-APC and NK-B cell colocalization
HELPER AND HUMORAL
CYTOTOXIC AND HUMORAL
CD4
CD4
B
B
CD4
B
B
DC
NK
NK
CD4
CD8
B
CD8
B
CD4
NK
CD8
DC
